# Delayed Signs and Symptoms of Extended Release Guanfacine Overdose in Two Adolescent Patients: Implications of Monitoring on the Psychiatry Unit

**DOI:** 10.1155/2022/2149301

**Published:** 2022-03-25

**Authors:** Sanskriti Mishra, Julia Shekunov, Della J. Derscheid, Elizabeth A. Canterbury, Jonathan G. Leung

**Affiliations:** Mayo Clinic, Rochester, MN, USA

## Abstract

Guanfacine is a selective alpha-2a adrenoreceptor agonist that with overdose can cause symptoms ranging from mild sedation to coma, respiratory depression, hyporeflexia, hypotonia, bradycardia, and hypotension. Despite a well-defined and predictable toxidrome, variations can be seen based on multiple factors including age, quantity ingested, organ functions, coingestions, time since ingestion, and specific dosage form. Here, we describe two cases of delayed presentation of extended release guanfacine toxicity and highlight the variations encountered in the toxidrome presentation. These cases bring to attention the importance of maintaining a high suspicion for such atypical presentations, keeping in mind the limitations of managing these complications on an inpatient psychiatric unit.

## 1. Introduction

Clinical manifestations following an overdose of medication are generally well predicted based on known pharmacologic properties. Yet, several factors may complicate the predicted onset, duration, and severity of clinical signs and symptoms following an overdose. These factors may include but are not limited to age, comorbidities, quantity of medication, metabolic pathway, interactions through coingestion, elapsed time to overdose detection, and specific dosage form of the medication. Following acute patient management after an overdose, the ability to transfer a patient from a nonpsychiatric medical setting to a psychiatric setting is determined by medical evaluation typically with input from poison control centers. However, residual toxicities or a delayed toxidrome presents potential safety issues in a psychiatric inpatient unit that may have limited immediate medical resources or be physically distant from direct access to emergent medical care. Two cases of delayed toxicity from guanfacine extended release (ER) are discussed along with the broader safety implications of toxicity presenting after admission to a psychiatric setting. Guanfacine is a centrally acting antihypertensive agent which acts as a selective alpha-2a adrenoreceptor agonist. In the pediatric population, guanfacine extended release has FDA approval for children ages 6-17 for treating attention deficit hyperactivity disorder (ADHD) as a monotherapy or in conjunction with a stimulant medication. Other non-FDA-approved indications for its use include Tourette's syndrome, anxiety, impulsivity, disruptive behaviors, and intrusive PTSD symptoms.

## 2. Cases

### 2.1. Case 1

An adolescent, weighing 54 kg, with a history of anxiety and depression was brought to the emergency department (ED) by family after an intentional overdose of four tablets of 2 mg guanfacine ER and three tablets of 20 mg escitalopram. The patient had one past suicide attempt, no prior psychiatric hospitalizations, and was not engaged in outpatient psychotherapy at the time of presentation. The patient completed a partial hospitalization program two months prior to the current presentation where they were prescribed escitalopram 20 mg daily for management of depression and anxiety and guanfacine extended release (ER) 2 mg daily to target symptoms of anxiety.

In the ED Hospital Day (HD) 1, postingestion time (PIT) of 1 hour, the patient was asymptomatic. Systolic blood pressure in the ED reached a nadir of 94 mmHg, and the patient received 2 liters of 0.9% sodium chloride intravenously. A complete blood count (CBC), basic metabolic panel, urinalysis, and electrocardiogram (ECG) were normal. Serum acetaminophen, salicylate, ethanol, urine pregnancy, urine toxicology screen, and SARS-CoV-2 testing were negative. Based on recommendations from the local poison control center, the patient was transferred to the pediatric intensive care unit (PICU) for monitoring.

Upon arrival to the PICU (HD 2, PIT 5 hours), the patient remained asymptomatic, hemodynamically stable, alert, and oriented. During early morning assessment by the nursing staff (HD 2, PIT 9 hours), the patient continued to deny symptoms suggestive of medication ingestion. Psychiatric consultation recommended admission to an inpatient psychiatric unit. In the absence of any new or emerging signs or symptoms, the patient was transferred from the PICU to the psychiatric unit (HD 2, PIT 15 hours).

One hour later, during the psychiatric nursing admission assessment (HD 2, PIT 16 hours), the patient was noted to be drowsy and observed with their eyes half open and needing to rest an arm on the sink of the admission room due to dizziness. Following the admission assessment, the patient relocated to their room, laid down, and quickly fell asleep. As nursing staff followed up on symptoms, the patient fell asleep at one point, midsentence. An end-of-shift pass-off and the arriving nurse's start-of-shift patient assessment were promptly conducted due to the patient's symptoms. At this time, the patient's reassessed vital signs revealed a blood pressure of 82/28 mmHg and heart rate in the upper 40s (HD 2, PIT 17 hours). An ECG demonstrated bradycardia with premature atrial complexes and a normal QTc. Given these findings and the patient's inability to remain awake, the rapid response team (RRT) was called. The RRT responds to nonadvanced cardiovascular life support medical concerns. The team recommended transfer of the patient to the ED for evaluation and possible readmission for monitoring.

In the ED (HD 2, PIT 20 hours), the patient was noted to be drowsy but arousable. The patient continued to be bradycardic with heart rate in the upper 40s and systolic blood pressure in the 90s. Repeat CBC, kidney functions, electrolytes, and thyroid function tests were normal, along with negative serum salicylate, acetaminophen, ethanol level, and urine toxicology screens. Notably, the patient was found to be in possession of an electronic nicotine delivery system (ENDS) in the ED. Given the unclear cause of ongoing bradycardia with hypotension, the patient was readmitted to the PICU for hemodynamic monitoring. Cardiology evaluation recommended conservative management with monitoring and no further testing. Almost PIT 40 hours, the patient remained hypotensive and bradycardic despite ongoing fluid administration. The patient consistently denied any additional ingestions while in the hospital and reaffirmed the initial stated quantity of ingestion of the tablets. On day two of PICU readmission (HD 4, PIT 60 hours), the patient's drowsiness and dizziness improved, with ability to ambulate without assistance. The patient was oriented to person, place, and time and hemodynamically stable. After transfer back to the inpatient psychiatric unit, there was no further hemodynamic compromise. The patient appropriately engaged in individual and group programming and began fluoxetine which was tolerated well. With reported improvements in both mood and anxiety, the patient was discharged after five days of inpatient psychiatric hospitalization (HD 9).

### 2.2. Case 2

An adolescent, weighing 90.1 kg, with a history of reactive attachment disorder, developmental speech and language disorder, learning disorder, anxiety, depression, and chronic behavioral difficulties without any prior inpatient psychiatric hospitalizations or suicide attempts, attending partial hospitalization program at the time of current presentation was brought to the ED via emergency medical services after intentional ingestion of forty-five tablets of 2 mg guanfacine ER and unknown quantities of loratadine and melatonin. Psychotropic medications prescribed prior to admission included citalopram 20 mg daily and bupropion extended release 150 mg daily for depression and anxiety and guanfacine ER 2 mg at bedtime for impulsivity.

In the ED (HD 1, PIT 1 hour), the patient was asymptomatic, alert, oriented, and normotensive, with a heart rate of 58 beats per minute. Complete blood count, basic metabolic panel, thyroid stimulating hormone, and ECG were unremarkable. Serum acetaminophen, salicylate, ethanol, urine pregnancy, and SARS-CoV-2 tests were negative. Based on recommendations from poison control, the patient was monitored in the ED for 5 hours and subsequently admitted to inpatient psychiatric unit.

Upon arrival to the inpatient psychiatric unit (HD 2, PIT 5.5 hours), the patient remained asymptomatic and hemodynamically stable. On HD 2, PIT 10 hours, the patient became hypertensive with systolic pressure ranging from 125 to 160 mmHg and diastolic pressure ranging from 87 to 107 mmHg. On HD 2, PIT 13 hours, the patient described feeling dizzy, nauseated, and diaphoretic with a blood pressure of 82/51 mmHg. After breakfast, blood pressure normalized, and nausea and dizziness subsided. Rapid response team (RRT) was called 2 hours later (HD 2, PIT 15 hours) as the patient reported dizziness, headache, and double vision and was observed to have slurred speech. Orthostatic vital signs revealed a blood pressure of 152/90 mmHg supine and 102/84 mmHg standing. During the RRT evaluation, systolic blood pressure ranged from 176 to 61, and diastolic ranged from 112 to 46, with heart rate of 50-60 BPM. The patient was transported to the ED for further evaluation.

In the ED (HD 2, PIT 17 hours), labs showed hemoconcentration (hematocrit 49.8%), normal basic metabolic panel, and EKG with sinus bradycardia. The patient received a bolus of 1 liter of normal saline. Poison control was contacted again who believed this to be a delayed effect of the guanfacine ER ingestion. The patient had access to bupropion and citalopram. However, these medications were accounted for, and the presentation was inconsistent with their toxidromes. The patient was subsequently admitted to the PICU for cardiovascular monitoring and stabilization.

In the PICU (HD 2, PIT 23 hours), urine drug screen was negative and prescription and over-the-counter drug screen was positive for bupropion and citalopram which were home medications. On HD 2, PIT 25 hours, the patient continued to have sustained hypertension up to 165/128 mmHg, requiring a dose of 15 mg intravenous hydralazine with improvement. On HD 3, PIT 32 hours, the patient had an orthostatic syncopal episode for 15 seconds before regaining consciousness without intervention. The patient was normotensive but bradycardic (58 BPM). EKG showed sinus bradycardia with normal QTc. Serum electrolytes were within normal limits. The following morning (HD 3, PIT 38 hours), the patient was noted to be lethargic, groggy, drowsy, and unable to hold a coherent conversation. Given hemodynamic stability, the patient was transferred from the PICU to the pediatric hospital service. In the evening, the patient reported orthostatic dizziness without any syncopal episode. Overnight (HD 4, PIT 57 hours), the patient became hypotensive (94/43 mmHg) with heart rate in 60s-70s BPM and received a 1-liter bolus of normal saline. In the morning, the patient was alert and oriented and able to hold a coherent conversation, denied dizziness, and tolerated oral diet. The patient continued to remain hypotensive throughout the day, and in the evening (HD 4, PIT 72 hours), blood pressure decreased to 78/38 mmHg requiring continuous normal saline infusion. The subsequent day (HD 5, PIT 88 hours), the patient was noted to be hemodynamically stable and was transferred back to the inpatient psychiatric unit.

On the inpatient psychiatric unit (HD 5, PIT 92 to 97 hours), the patient again became hypotensive with blood pressures 115/35 mmHg supine and 76/40 mmHg standing. The patient was asymptomatic except for a headache which responded well to ibuprofen. Vital signs improved with increased oral fluid intake. By HD 7, PIT 134 hours, vital signs had stabilized with no further hemodynamic compromise. The patient participated well in unit programming and with continued improvements in mental health symptoms, without reinitiation of psychotropic medications, was discharged home after five days of inpatient psychiatric hospitalization (HD 9).

## 3. Discussion

Two adolescents were transferred to an inpatient psychiatric unit having limited signs or symptoms of toxicity from an intentional ingestion of guanfacine ER. However, 13-17 hours after ingestion, hypotension and somnolence developed, necessitating the need for a higher level of care for an extended period. While there was a coingestion with escitalopram in the first case, there were no signs or symptoms that would be common of serotonin syndrome. It is possible that a 60 mg dose of escitalopram could potentially contribute to altered mental status or sedation in the first several hours after ingestion, but clinically, the somnolence followed by persistent bradycardia and hypotension was likely the result of guanfacine ER. One consideration is that what was believed to be an ENDS could have contained other substance(s) but there was no further investigation at the time. A pharmacokinetic interaction potentiating the toxidrome is also not likely as guanfacine is primarily metabolized via CYP450 3A4. Escitalopram is primarily a substrate of CYP450 2C19 and to a lesser degree CYP450 3A4. Escitalopram weakly inhibits only CYP450 2D6. Pharmacogenomic testing had not been completed for review. In the second case, known coingestions included loratadine and melatonin. Loratadine is associated with sedation, mild hemodynamic changes, and rarely dysrhythmias, edema, or seizures. Melatonin ingestions may result in sedative effects but typically without significant sequela.

Pharmacodynamically, guanfacine results in lowered blood pressure via agonism at alpha-2a adrenoreceptor [1]. This mechanism causes reduced sympathetic outflow and a subsequent decrease in vasomotor tone and heart rate [2]. Following an ingestion of guanfacine, the lack of symptoms is not uncommon, but when clinical toxicity occurs, mild sedation to coma, respiratory depression, hyporeflexia, hypotonia, bradycardia, and hypotension are possible [2]. After a large ingestion, guanfacine may paradoxically cause brief hypertension via postsynaptic agonism.

Guanfacine is available as both an immediate release (IR) and ER formulation. The IR product results in a peak serum concentration after 1-4 hours of taking a usual therapeutic dose. Peak concentrations are delayed up to 5 hours for the ER formulation. Due to differences in bioavailability, the same strength of ER guanfacine, as compared to the immediate release product, results in a 60% lower peak serum concentration and 43% lower drug exposure [1]. The half-life of guanfacine has been reported to range from 10 to 30 hours and can vary based on age and kidney and liver function. In general, it can be predicted that in overdose situations, ER guanfacine may result in delayed and prolonged signs and symptoms, noting that pharmacokinetics can be altered in overdose.

Literature predominantly confers the need for monitoring of patients in a (medical) healthcare setting when symptoms of toxicity are present. The presentation of only lethargy is important as this may indicate the potential for delayed hypotension or bradycardia. Tachycardia should also be considered a prodromal sign to possible hypotension. Based on available guidelines and in line with the conclusions of Winograd et al. [2], (1) patients 0-12 years of age with guanfacine exposures should be monitored in a medical setting or any age if the ingestion is twice prescribed dose, (2) those of any age should present to a medical setting if symptoms are present or if the ingestion was intentional, (3) symptomatic patients should be monitored for at least 24 hours, or (4) those meeting criteria for home observation should have follow-up via telephone call. In the cases presented, the patients were initially admitted for monitoring based on the dose and unclear circumstances of the intent of the ingestion. With a lack of symptoms that should have been seen if there would have been concerns, both patients were transferred to an inpatient psychiatry unit. Unfortunately, both subsequently developed somnolence, hypotension, and bradycardia requiring an additional monitoring in the PICU.

In reviewing the literature, 13 articles were found related to guanfacine overdose and toxicity. This included seven case reports and six studies summarized in Table 1. Cases describe signs and symptoms of guanfacine toxicity that were delayed, prolonged, or both [3–10].

These cases highlight important safety considerations, potential quality improvement initiatives, and education opportunities. First, the recognition of common toxicities of medications is important for the entire psychiatric team, including nursing staff who have significant ongoing interactions with a patient. It was the awareness of a nurse in the presented cases that led to the RRTs being called and ultimate transfers back to the PICU. While cases of delayed and unexpected toxicities are not rare in the literature, no reports, quality initiatives, or education was identified that discussed the importance of psychiatric staff education on expected toxidromes following medication ingestion at doses greater than prescribed. As such, it is important that all staff have a basic understanding of common signs and symptoms that may present either acutely or delayed following an ingestion of medication. One initiative for our child and adolescent inpatient psychiatric unit staff, through collaboration with emergency department and media support service staff, was the creation of educational materials, including a quick reference guide of various toxidromes (Supplemental Table). The quick reference guide titled, *Overview of Commonly Encountered Toxidromes*, was readily received by the inpatient psychiatric staff. Clinically, the table was deemed important as a reference not only for guanfacine overdoses but also for other overdose medications commonly used by youth. The recommended monitoring parameters in this tool support bedside clinical assessment, tracking of symptom trends, and thus early identification of worrisome patient status. The table is readily accessible to staff in the unit medication room as well as the unit online resource.

## 4. Conclusion

These two cases highlight the potential for delayed signs and symptoms following guanfacine ingestions. All staff caring for patients following management of medication ingestion should be familiar with the potential signs and symptoms of toxicity. There are opportunities and benefits in reviewing various toxidromes with staff in the inpatient psychiatric setting, noting that there can be delayed presentations.

## Figures and Tables

**Table 1:** Review of literature.

Author, year	Type	Comments
Kolarich, 2019 [10]	Case report	17-year-old ingested 189 mg ER guanfacine and 340 mg of olanzapine. Transferred from another facility (timing not described), presented with bradycardia, hypotension, somnolence, and orientation only to self over the first 24 hours. On day 4, became fully alert and oriented with bradycardia resolving. Between days 4 and 6 experienced sinus pause with forcefully drinking fluid, which resolved.
Bridwell, 2021 [9]	Case report	17-year-old presented 2 hours following 80 mg ingestion of ER guanfacine. Home dose was prescribed as 2 mg per day. Presented sleepy but arousable with sinus bradycardia. Respiratory distress developed necessitating intubation and heart failure with reduced EF of 30%. Extubated on day 5.
Fein, 2013 [8]	Case report	12-year-old presented 18 hours following 12 mg ingestion of ER guanfacine. Home dose was prescribed as 4 mg per day. Presented with sedation and unable to stand without assistance. HR 45, BP 140/80. BP peaked at 170/116 and IV nicardipine started for 3 hours. 36 hours after ingestion, symptomatic hypotension developed 70/26 mmHg with SBP < 90 mmHg up to 85 hours postingestion.
Minns, 2010 [7]	Case report	16-year-old with ingestion of 25 mg of IR guanfacine. Prescribed dose was 1 mg per day. Reported to parents 8 hours postingestion, BP at that time 160/120 and taken to ED. ED discharged 2 hours later with BP and HR stable. At home had a syncopal episode (BP 97/57) and returned to ED where BP was 67/30 standing, QTc 593 msec. Vital signs normalized and symptoms of orthostasis ceased by 60 hours after ingestion.
Van Dyke, 1990 [5]	Case report	2-year-old presented 1.5 hours after 4 mg guanfacine exposure. Initial BP was 100/60 mmHg and HR 83 bpm. Somnolence and diaphoresis noted. 18 hours postexposure SBP and HR decreased as low as 58 mmHg and 66 bpm, respectively. Discharged without intervention, 24 hours after admission.
Walton, 2014 [4]	Case report	8-year-old received an accidental second dose of 3 mg ER guanfacine at school. Somnolence, hypotension, and bradycardia were noted 3 hours after. Improvements by 18 hours postexposure with monitoring needed until 45 hours after exposure.
Fontane, 2013 [3]	Case report	2-year-old presented approximately 12 hours postingestion of nearly 24 mg guanfacine. Somnolence, bradycardia, mild hypertension, and miosis reported. Monitored in the PICU for approximately 24 hours and discharged next day.
Baumgartner, 2021 [11]	Single center, retrospective chart review	Most guanfacine ingestions (*n* = 19) were patients aged 13-18 (53%) as a single substance ingestion (58%). 95% were admitted and 32% with at least 1 day in the ICU. Intravenous fluids were the primary intervention required. No deaths were directly attributed to guanfacine.
Winograd, 2019 [2]	Review of a nation poison control center database (2000-2016)	10,927 of pediatric guanfacine exposure cases identified in the National Poison Data System. Common signs and symptoms included: drowsiness (39%), bradycardia (15.5%), and hypotension (10.3%). Duration of effects between 8 and 24 hours occurred in 44.2% of cases. Of all symptomatic cases, 80% had resolution within 24 hours. One death reported. 50.3% of cases were asymptomatic. The authors recommend evaluation at a healthcare facility for children 0–12 years old exposed to guanfacine, or any age if the ingestion is twice prescribed dose, and for all pediatric patients with intentional ingestion. Symptomatic patients should be monitored for at least 24 hours postexposure and via telephone follow-up for asymptomatic cases.
McGrath, 2002 [12]	Review of a nation poison control center database (1993-1999)	870 cases were reviewed with most being children < 6 years of age (54.9%). 62.8% of exposures resulted in no symptoms. Most common symptoms were drowsiness (76.8%), bradycardia (30%), and hypotension (25.8%). 19.9% of cases had ICU admissions and no deaths. Delay or duration of symptoms was not discussed. The authors concluded, pediatric patients with sedation should be observed for up to 24 hours due to the risk of developing delayed toxicities such as coma, bradycardia, and hypotension.
Levine, 2013 [13]	Multicenter, retrospective chart review	Review of ingestion cases and cost analysis related to commonly used medication for ADHD. Data of all medications pooled, so unable to specifically describe outcomes with guanfacine individually. In two separate cohorts, guanfacine accounted for 5.3% of cases (2000-2002) and 8.7% of cases (2009-2010).
Snyder, 2020 [14]	Single center, retrospective chart review	Review of guanfacine exposures in children less than 6 years of age, noting an increase in this population. There were 28 cases in this age group 11/2014–12/2019. Mean exposure was 2.5 mg of which 39.3% had prescriptions for guanfacine. 53.6% of cases were asymptomatic. Most reported symptom was lethargy (84.6%). The authors disagree with Winograd that all pediatric exposures 0-12 years of age require referral to a healthcare facility. Recommend individual cases should be assessed by the poison center and referred based on patient specific factors.
Wang, 2014 [15]	Review of a nation poison control center database (2000-2011)	The authors reviewed alpha-2 agonist exposures as a class. Guanfacine accounted for 22% of the 27,825 exposures. Most common symptoms in guanfacine exposures were drowsiness (28.7%), bradycardia (8.1%), and hypotension (5.8%). 12 patients developed coma. There were no deaths. Timing was not discussed.

## Supplementary Materials

Supplementary Materials The quick reference guide titled, *Overview of Commonly Encountered Toxidromes*, was created through collaboration with the emergency department pharmacist and media support service staff. It is currently in use as an educational tool for psychiatric unit staff to quickly review potential signs and symptoms associated with various toxidromes.
