# Supplementary material for: Delayed Signs and Symptoms of Extended Release Guanfacine Overdose in Two Adolescent Patients: Implications of Monitoring on the Psychiatry Unit
Source: Case Rep Psychiatry. 2022 Mar 25;2022:2149301. doi: 10.1155/2022/2149301 (PMC8975677; doi:10.1155/2022/2149301)
Supplement: Supplementary Materials — The quick reference guide titled, Overview of Commonly Encountered Toxidromes, was created through collaboration with the emergency department pharmacist and media support service staff. It is currently in use as an educational tool for psychiatric unit staff to quickly review potential signs and symptoms associated with various toxidromes. [file 2149301.f1.pdf]

Supplementary Table 2. Overview of Commonly Encountered Toxidromes*, **

Legend									
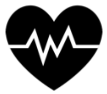	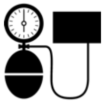	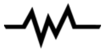	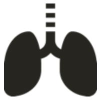	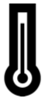	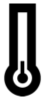	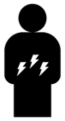	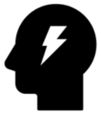	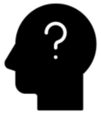	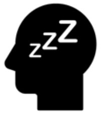
HR	BP	ECG changes	RR	↑temp	↓temp	Hyper-active bowel sounds	Agitation	Confused	Lethargic/ somnolent

**Class/Medication**	**Common overdose symptoms**	**Common Signs, Symptoms, and Monitoring Parameters**						**Notes on monitoring duration**
		**HR/BP**	**RR**	**Temp**	**Bowels**	**Mental Status**	**Other**	
**Acetaminophen**	• Widely variable based on amount and time from ingestion; may be symptomatic or only develop nausea, vomiting and abdominal pain early.<br>• LFTs may rise after 12 hours after ingestion with few signs or symptoms, peaking 2-3 days later. Severe symptoms: those of liver failure, including coagulopathy and encephalopathy, with renal dysfunction and metabolic disturbances.	No change	No change	No change	No change	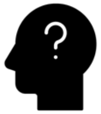 (usually after >48 hours in severe cases)	Nausea, vomiting<br>Abdominal pain<br>LFTs, CBC, INR	• Severity dependent; hepatic or renal failure would be evident within 72 hours
**Aspirin (Note: Aspirin has a distinct toxidrome. Other agents that are NSAIDS (e.g. ibuprofen) are fairly benign in overdose. In massive overdose they may produce drowsiness and metabolic acidosis may rarely be seen)**	• **Mild-moderate**: GI upset, tinnitus, tachypnea<br>• **Severe**: Metabolic acidosis, diaphoresis, altered mental status, seizures, coma, pulmonary edema	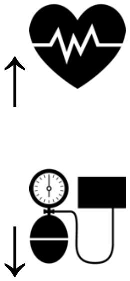	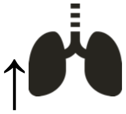	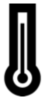	No change or ↓	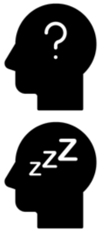	Tinnitus<br>Acid/base abnormality<br>Vomiting<br>Seizures<br>Neuroglycopenia	• Dependent on salicylate levels; may need initial monitoring of about 24 hours

Legend

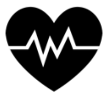	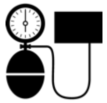	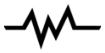	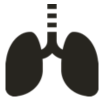	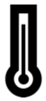	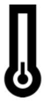	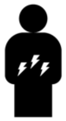	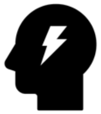	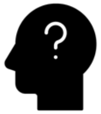	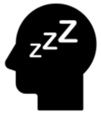
HR	BP	ECG changes	RR	↑temp	↓temp	Hyper-active bowel sounds	Agitation	Confused	Lethargic/ somnolent

Class/Medication	Common overdose symptoms	Common Signs, Symptoms, and Monitoring Parameters						Notes on monitoring duration
		**HR/BP**	**RR**	**Temp**	**Bowels**	**Mental Status**	**Other**	
**Alpha-1 antagonists** (e.g. prazosin)	• **Mild-moderate**: hypotension, tachycardia, GI symptoms, mild sedation<br>• **Severe**: Hypotension, ECG changes to TdP	↑ 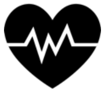<br>↓ 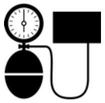<br>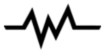	No change	No change	No change	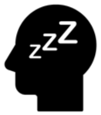<br>(mild)	Rare: Seizures, agitation, priapism	• Generally 6-12 hours
**Alpha-2 agonists** (e.g. clonidine, guanfacine)	• **Mild-moderate**: CNS depression, bradycardia, QTc prolongation, initial transient hypertension followed by hypotension<br>• **Severe**: CNS depression, cardiac events should be anticipated, hypotension which may be delayed and prolonged.	↓ 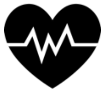<br>↓↑ 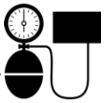<br>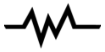	Variable	No change	No change or ↓	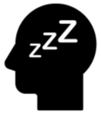<br>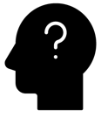	May see initial and transient increases to blood pressure and a delayed hypotension after several hours	• Onset of symptoms within 30-90 minutes<br>• Depends on dosage form 4 hours (IR) to 14 hours (ER)<br>• Prolonged presentation possible with extended-release forms (24-72 hours)

Legend									
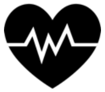	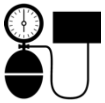	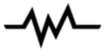	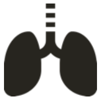	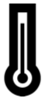	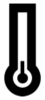	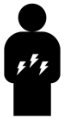	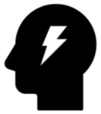	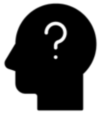	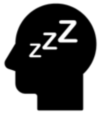
HR	BP	ECG changes	RR	↑temp	↓temp	Hyper-active bowel sounds	Agitation	Confused	Lethargic/ somnolent

Class/Medication	Common overdose symptoms	Common Signs, Symptoms, and Monitoring Parameters						Notes on monitoring duration
		**HR/BP**	**RR**	**Temp**	**Bowels**	**Mental Status**	**Other**	
**Anticholinergic agents** (e.g. benztropine, diphenhydramine)	• **Mild-moderate**: Somnolence, anticholinergic, tachycardia, hypertension, GI symptoms, mental status changes<br>• **Severe**: Delirium, agitation, psychosis, hallucinations, seizures, hyperthermia, coma, ECG changes	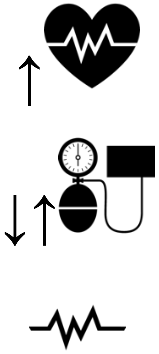	No change or ↑	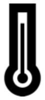	↓	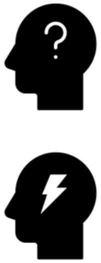	Hallucination<br>Skin flushing<br>Urinary retention<br>Seizures<br>Gastrointestinal hypomotility	• Agent and dosage form specific, at least 6 to 8 hours
**Antipsychotics**, presentation represents general class effects, presentation may be agent specific (e.g. risperidone, haloperidol, quetiapine, paliperidone, aripiprazole, olanzapine)	• **Mild-moderate**: Anticholinergic effects, sedation, hypotension mild tachycardia, QTc prolongation<br>• **Severe**: CNS depression, anticholinergic poisoning, seizures, tachycardia, hypotension, QTc prolongation, respiratory depression, **NMS**	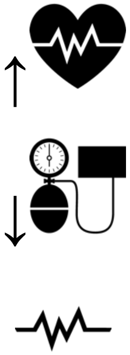	↓ 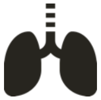	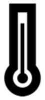	No change or ↓	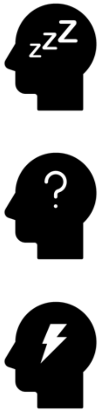	Seizures<br>Orthostatic hypotension	• Variable based on agent, at least 6-8 hours<br>• Monitoring for NMS may be warranted for up to 24-72 hours<br>• Do not restart antipsychotics until after 14 days of symptom resolution if NMS occurs

Legend

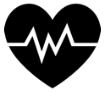	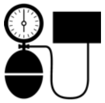	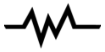	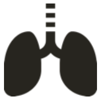	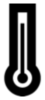	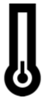	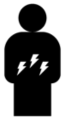	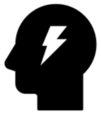	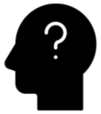	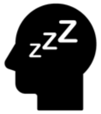
HR	BP	ECG changes	RR	↑temp	↓temp	Hyper-active bowel sounds	Agitation	Confused	Lethargic/ somnolent

Class/Medication	Common overdose symptoms	Common Signs, Symptoms, and Monitoring Parameters						Notes on monitoring duration
		**HR/BP**	**RR**	**Temp**	**Bowels**	**Mental Status**	**Other**	
**Antihyperglycemics**								
Metformin	• **Mild-moderate**: GI symptoms, malaise, and myalgia<br>• **Severe:** lactic acidosis, mental status changes, hypotension, and renal failure	Changes generally only in severe cases	Changes generally only in severe cases	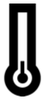 Hypothermia possible in severe cases	No change or ↑	Changes generally only in severe cases	Glucose monitoring (hypoglycemia unlikely)<br><br>GI Symptoms<br><br>Serum creatinine	• Dosage form specific, at least 8-12 hours
Sulfonylureas	• **Mild-moderate**: More directly affects glucose and toxicity is usually secondary to hypoglycemia<br>• **Severe:** CNS symptoms resulting from hypoglycemia including seizures, altered mental status, delirium, focal neurologic effects, and coma may result. Potential for dysrhythmias	↑ 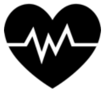	Changes generally only in severe cases	No change	No change	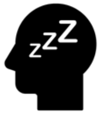 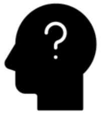 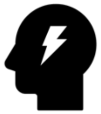 Usually secondary to hypoglycemia	Glucose monitoring	• Agent and dosage form specific, at least 18 hours (IR) to 24 hours (ER)<br>• Hypoglycemia may be present for up to 72 hours

Legend									
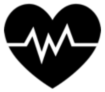	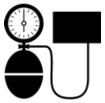	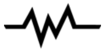	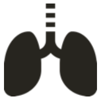	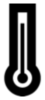	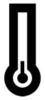	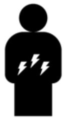	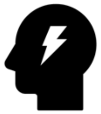	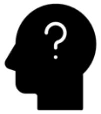	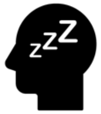
HR	BP	ECG changes	RR	↑temp	↓temp	Hyper-active bowel sounds	Agitation	Confused	Lethargic/ somnolent

**Class/Medication**	**Common overdose symptoms**	**Common Signs, Symptoms, and Monitoring Parameters**						**Notes on monitoring duration**
		**HR/BP**	**RR**	**Temp**	**Bowels**	**Mental Status**	**Other**	
**Benzodiazepines** (e.g. lorazepam, clonazepam, diazepam, alprazolam)	• **Mild-moderate**: CNS/ respiratory depression (note: Coingestion of other CNS depressants including alcohol increases risk of effects dramatically)<br>• **Severe**: Respiratory depression/arrest. Effects are variable based on specific agent and occurrence of a co-ingestion	↓ 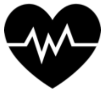<br>↓ 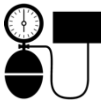<br>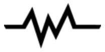	↓ 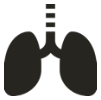	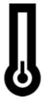	↓ or Ø	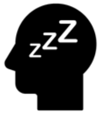<br>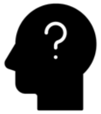	Ataxia<br>Hyporeflexia	• Agent specific, monitoring for as long as CNS/ respiratory depression is evident
**Beta-blockers** (e.g. propranolol, metoprolol, atenolol)	• **Mild-moderate**: bradycardia, hypotension<br>• **Severe**: ECG change/conduction delays, hypotension, renal failure, coma and cardiopulmonary arrest, bronchospasm. Propranolol easily crosses the into the CNS and may cause more sedation/ seizures	↓ 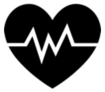<br>↓ 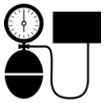<br>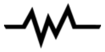	Dyspnea, bronchospasm (especially with history of asthma/COPD)	No change	No change	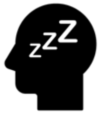<br>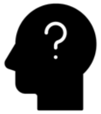	Hypoglycemia<br>Seizures<br>CNS depression	• Agent and dosage form specific, at least 6 hours or longer with ER products

Legend									
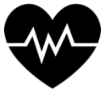	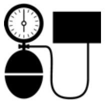	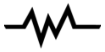	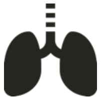	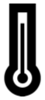	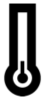	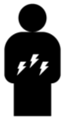	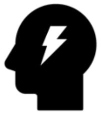	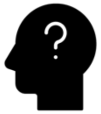	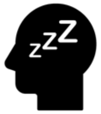
HR	BP	ECG changes	RR	↑temp	↓temp	Hyper-active bowel sounds	Agitation	Confused	Lethargic/ somnolent

**Class/Medication**	**Common overdose symptoms**	**Common Signs, Symptoms, and Monitoring Parameters**						**Notes on monitoring duration**
		**HR/BP**	**RR**	**Temp**	**Bowels**	**Mental Status**	**Other**	
**Mood Stabilizers (Carbamazepine, divalproex sodium/valproic acid, lamotrigine, lithium)**								
Carbamazepine	• **Mild-moderate**: nystagmus, ataxia, hyperreflexia, CNS depression, dystonia, tachycardia, tremor anticholinergic effects<br>• **Severe**: coma, seizures, respiratory depression, renal failure, cardiac toxicity, hyponatremia rhabdomyolysis, hepatotoxicity	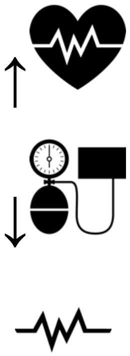	Depends on severity 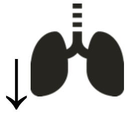	No change	↓	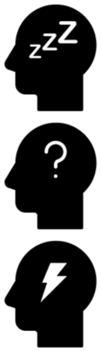	LFTs, CBC, electrolytes<br><br>Anticholinergic effects<br><br>Seizure<br><br>Rash<br><br>Motor symptoms/gait	• Dosage form specific, minimum of 12 hours (IR) or up to 96 hours (ER)<br>• Unpredictable absorption, can check serum levels to ensure declining concentrations
Divalproex sodium, valproic acid	• **Mild-moderate**: lethargy/sedation, mental status changes, GI symptoms, hyperammonemia, transaminitis, tremor<br>• **Severe**: CNS/respiratory depression, ECG changes, tachycardia, hypotension, hypernatremia, hepatotoxicity, hyperammonemia<br>• **Other**: Delayed bone marrow suppression, pancreatitis	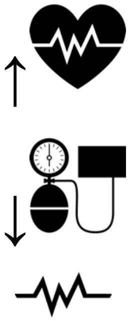	Depends on severity 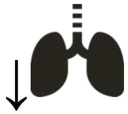	No change	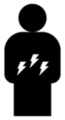	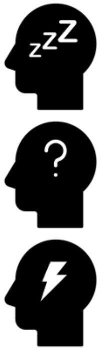	LFTs, CBC, electrolytes<br><br>Nausea, vomiting, diarrhea<br><br>Motor symptoms/gait	• Dosage form specific, at least 6 hours (IR) or at least 12-24 hours (ER) or longer if symptomatic<br>• Valproic acid levels can be monitored to ensure declining concentrations

Legend

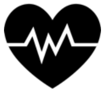	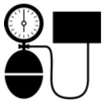	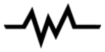	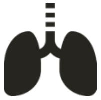	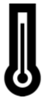	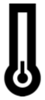	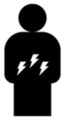	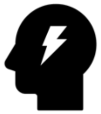	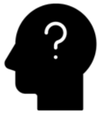	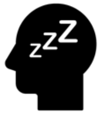
HR	BP	ECG changes	RR	↑temp	↓temp	Hyper-active bowel sounds	Agitation	Confused	Lethargic/ somnolent

**Class/Medication**	**Common overdose symptoms**	**Common Signs, Symptoms, and Monitoring Parameters**						**Notes on monitoring duration**
		**HR/BP**	**RR**	**Temp**	**Bowels**	**Mental Status**	**Other**	
Lamotrigine	• **Mild-moderate**: GI ADEs, CNS depression, tremor, xerostomia, vision changes, gait changes, transaminitis<br>• **Severe**: ECG changes, electrolyte abnormalities, oculogyric crisis, seizures, rhabdomyolysis, coma encephalopathy, and respiratory depression<br>• **Other**: seizure, hypersensitivity reactions that may be delayed (3-5 weeks)	Variable 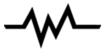	Depends on severity 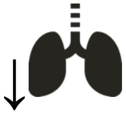	No change	No change	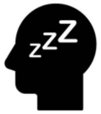 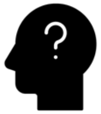 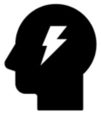	LFTs, CBC<br><br>Motor symptoms/gait<br><br>Rash	• Dosage form specific, at least 6-8 hours (IR) or up to12-24 hours (ER)
Lithium	• **Mild-moderate**: Nausea, vomiting, diarrhea, CNS depression, mental status changes, tremor, AKI, serotonergic toxicities, ECG changes (bradycardia, T-wave abnormalities)<br>• **Severe**: electrolyte abnormalities, acute kidney injury, seizure hyperthermia, coma, rigidity, myoclonus, serotonin syndrome. severe ECG changes, hypotension<br>• **Other**: sustained effects possible: cerebellar and cognitive dysfunction, EPS-like symptoms	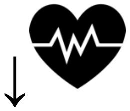 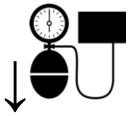 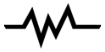	No change	No change	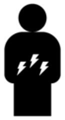	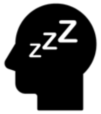 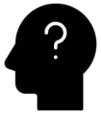 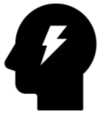	Serum creatinine, electrolytes<br><br>Nausea, vomiting diarrhea<br><br>Motor symptoms/gait<br><br>Serotonin like picture possible	• Dosage form specific; at least 6 hours (IR) or up to 12 hours (ER)<br>• Initial serum levels may not correlate well in acute ingestion

Legend

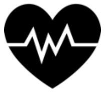	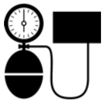	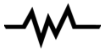	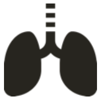	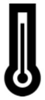	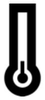	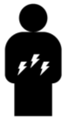	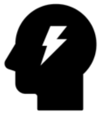	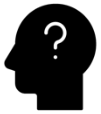	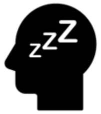
HR	BP	ECG changes	RR	↑temp	↓temp	Hyper-active bowel sounds	Agitation	Confused	Lethargic/ somnolent

Class/Medication	Common overdose symptoms	Common Signs, Symptoms, and Monitoring Parameters						Notes on monitoring duration
		HR/BP	RR	Temp	Bowels	Mental Status	Other	
**Opioids**	• **Mild-moderate**: Euphoria, drowsiness, constipation, nausea, vomiting, miosis, mild bradycardia, mild hypotension<br>• **Severe**: Respiratory depression, hypoxia, coma, bradycardia, prolonged QT, or acute lung injury. Potential for serotonergic effects (especially tramadol, fentanyl, meperidine)	↓ 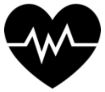<br>↓ 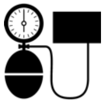<br>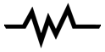	↓ 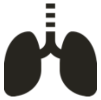	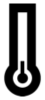	↓ or Ø	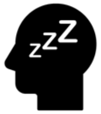	Pulmonary edema<br><br>Seizures (with some agents)<br><br>Rare: serotonin syndrome	4-6 hours unless:<br>• ER formulation (12 hours)<br>• Buprenorphine or fentanyl patch (6 to 12 hours)<br>• Buprenorphine (12 hours)
**Serotonergic agents** Antidepressants (e.g., SSRI, SNRI, TCA), opioid pain medications, various OTCs (e.g. dextromethorphan, St. John's Wort), list not inclusive **(Note bupropion is distinct in that it is more likely associated with seizures, agitation, hallucinations, tachycardia, mild hypertension)**	• **Mild-moderate**: Somnolence, dizziness, agitation, GI symptoms, tachycardia, palpitations, hypertension, elevated LTFs, and cutaneous vasodilation.<br>• **Severe**: CNS depression. serotonin syndrome (clonus, hyperreflexia, altered mental status, or hemodynamic instability), seizures	↑ 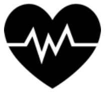<br>↑ 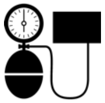<br>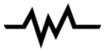	Depends on severity<br>↑ 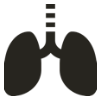	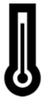	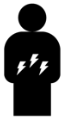	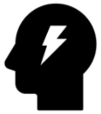	Clonus, muscle rigidity, hyperreflexia<br><br>Serum creatinine, creatinine kinase	• Presentation within hours<br>• Resolution in mild-moderate case within 24 to 48 hours.<br>• Do not restart serotonergic agents until after complete resolution of symptoms

**Legend**									
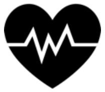	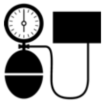	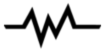	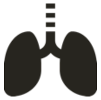	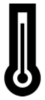	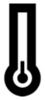	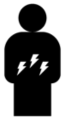	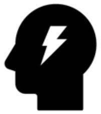	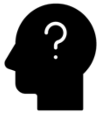	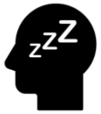
HR	BP	ECG changes	RR	↑temp	↓temp	Hyper-active bowel sounds	Agitation	Confused	Lethargic/ somnolent

Class/Medication	Common overdose symptoms	Common Signs, Symptoms, and Monitoring Parameters						Notes on monitoring duration
		HR/BP	RR	Temp	Bowels	Mental Status	Other	
**Sympathomimetic/ stimulants**	• **Mild-moderate**: tachycardia, hypertension, restlessness, agitation<br>• **Severe**: ECG changes, arrhythmias, hypertensive crisis, psychosis, hyperreflexia, serotonin syndrome	↑ 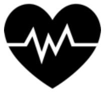<br>↑ 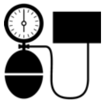<br>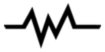	↑ 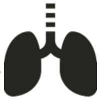	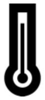	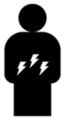	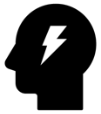	Tremors<br>Skin flushing, sweating<br>Hyperactivity<br>Seizures	• Dosage form specific, at least 6-8 hours (IR) or up to12-24 hours (ER)<br>• Many dosage forms exist with differing release mechanisms

*Content adapted from MN Poison Control guidelines, Hoffman R.S., & Howland M, & Lewin N.A., & Nelson L.S., & Goldfrank L.R.(Eds.), (2015). Goldfrank's Toxicologic Emergencies, 10e. McGraw Hill.

**Table used with permission of Mayo Foundation for Medical Education and Research

Abbreviations: ADE = adverse drug events, AKI = acute kidney injury, BP = blood pressure, CBC = complete blood count, COPD = chronic obstructive pulmonary disease, CNS = central nervous system, ECG = electrocardiogram, EPS = extrapyramidal symptoms, ER = extended release, GI = gastrointestinal, HR = heart rate, INR = international normalized ratio, IR = immediate release, LFTs = liver function tests, NMS = neuroleptic malignant syndrome, NSAIDs = non-steroidal anti-inflammatory drugs, OTC = over the counter medications, RR = respiratory rate, SNRI = serotonin and norepinephrine reuptake inhibitor, SSRI = selective serotonin reuptake inhibitor, TCA = tricyclic antidepressant, TdP = torsade de pointes, temp = temperature

Comments:

- Symptom and timing presentation may be variable based acute, acute-on-chronic, or chronic toxicity
- Note medication history and formulations that are delayed/extended-release product; reports exist of symptoms presenting as late as 24+ hours.
- Pharmacobezoars are the result of solid dosage forms conglomerating. They are rare but can significantly alter/delay/prolong presentation
- Always consider unknown co-ingestions
